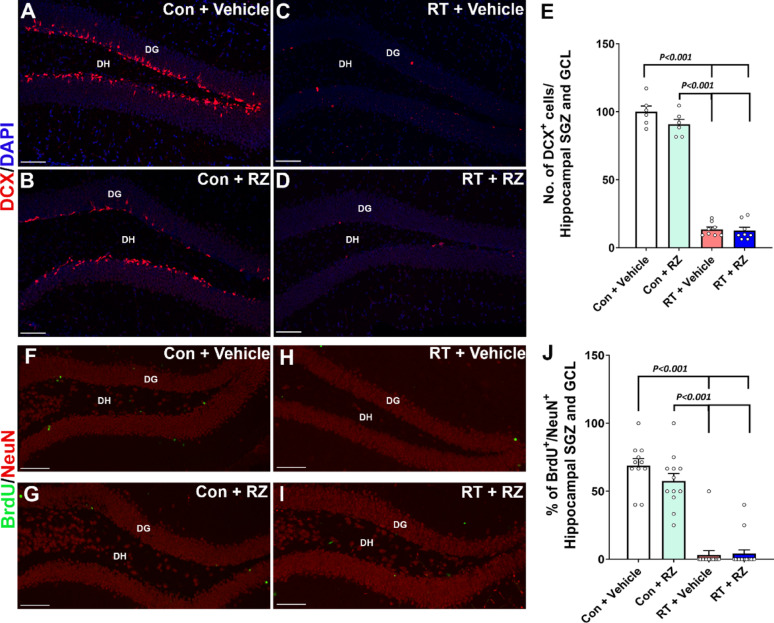# Correction: BDNF augmentation reverses cranial radiation therapy-induced cognitive decline and neurodegenerative consequences

**DOI:** 10.1186/s40478-025-02147-0

**Published:** 2025-11-10

**Authors:** Sanad M. El-Khatib, Arya R. Vagadia, Anh C. D. Le, Janet E. Baulch, Ding Quan Ng, Mingyu Du, Kevin G. Johnston, Zhiqun Tan, Xiangmin Xu, Alexandre Chan, Munjal M. Acharya

**Affiliations:** 1 Department of Anatomy and Neurobiology, School of Medicine, University of California, Irvine, USA; 2 Department of Radiation Oncology, School of Medicine, University of California, Irvine, USA; 3 Department of Clinical Pharmacy Practice, School of Pharmacy and Pharmaceutical Sciences, University of California, Irvine, USA; 4 Center for Neural Circuit Mapping, School of Medicine, University of California, Irvine, USA; 5 Department of Pharmaceutical Sciences, School of Pharmacy and Pharmaceutical Sciences, University of California, Irvine, USA

**Correction to: Acta Neuropathologica Communications (2024) 12:190** 10.1186/s40478-024-01906-9

In Figure 5F–I, of this article [1], the label color for BrdU-NeuN is swapped from green to red. The incorrect label is . The correct label should be: . For completeness and transparency, both incorrect and correct versions with updated figure legends are displayed below.

Incorrect Fig. 5
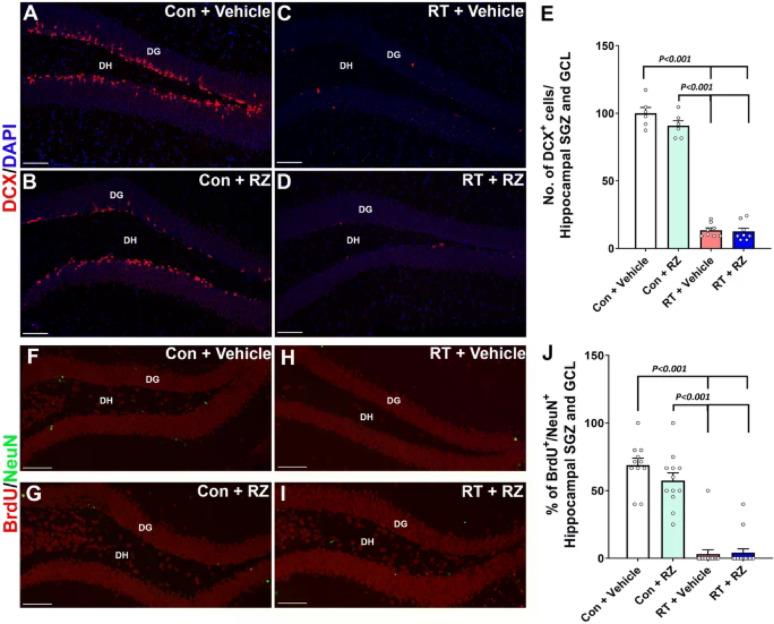


**Fig. 5** Riluzole treatment did not prevent cranial radiation-induced decline in neurogenesis. WT adult male mice received cranial RT (9 Gy) and treated with riluzole (RZ, 13 mg/kg) 48 h later in their drinking water for 6–7 weeks. Two weeks post-RT, mice were treated with BrdU and hippocampal neurogenesis was quantified using newly born neuron marker, doublecortin (DCX), and BrdU-NeuN dual-immunofluorescence staining in the hippocampal dentate gyrus (DG) sub-granular zone (SGZ) and molecular layer (ML) 6–7 weeks after the BrdU treatment. Cranial RT (RT + Vehicle) significantly reduced the number of DCX^+^ neurons (red, **A**–**D**) in the hippocampus compared with either Control + Vehicle or Control + RZ groups **E**. RT also significantly reduced neurogenesis, as indicated by the reduced percentage of BrdU^+^ cells (green) differentiating into the mature neurons (red, NeuN; **F**–**I**) in the RT + Vehicle group compared with either Control + Vehicle or Control + RZ groups (**J**). Riluzole treatment to the irradiated animals did not prevent the loss of DCX^+^ newly born neurons and the decline in dentate neurogenesis (BrdU^+^-NeuN^+^ dual-fluorescent cells). Data is presented as mean ± SEM (N = 6–16 mice per group). *P* values were derived from two-way ANOVA and Bonferroni's multiple comparisons test. Scale bars, 50 μm, (**A–D**) and (**F**–**I**)

Correct Fig. 5

**Fig. 5** Riluzole treatment did not prevent cranial radiation-induced decline in neurogenesis. WT adult male mice received cranial RT (9 Gy) and treated with riluzole (RZ, 13 mg/kg) 48 h later in their drinking water for 6–7 weeks. Two weeks post-RT, mice were treated with BrdU and hippocampal neurogenesis was quantified using newly born neuron marker, doublecortin (DCX), and BrdU-NeuN dual-immunofluorescence staining in the hippocampal dentate gyrus (DG) sub-granular zone (SGZ) and molecular layer (ML) 6–7 weeks after the BrdU treatment. Cranial RT (RT + Vehicle) significantly reduced the number of DCX^+^ neurons (red, **A**–**D**) in the hippocampus compared with either Control + Vehicle or Control + RZ groups **E**. RT also significantly reduced neurogenesis, as indicated by the reduced percentage of BrdU^+^ cells (green) differentiating into the mature neurons (red, NeuN; **F**–**I**) in the RT + Vehicle group compared with either Control + Vehicle or Control + RZ groups (**J**). Riluzole treatment to the irradiated animals did not prevent the loss of DCX^+^ newly born neurons and the decline in dentate neurogenesis (BrdU^+^-NeuN^+^ dual-fluorescent cells). Data is presented as mean ± SEM (N = 6–16 mice per group). *P* values were derived from two-way ANOVA and Bonferroni's multiple comparisons test. Scale bars, 50 μm, (**A–D**) and (**F**–**I**)